# Comparative Proteomic Analysis Provides Insight into the Key Proteins Involved in Cucumber (*Cucumis sativus* L.) Adventitious Root Emergence under Waterlogging Stress

**DOI:** 10.3389/fpls.2016.01515

**Published:** 2016-10-13

**Authors:** Xuewen Xu, Jing Ji, Xiaotian Ma, Qiang Xu, Xiaohua Qi, Xuehao Chen

**Affiliations:** Department of Horticulture, School of Horticulture and Plant Protection, Yangzhou UniversityYangzhou, China

**Keywords:** cucumber, waterlogging, hypocotyls, adventitious root, iTRAQ

## Abstract

Waterlogging is a common abiotic stress in both natural and agricultural systems, and it primarily affects plant growth by the slow oxygen diffusion in water. To sustain root function in the hypoxic environment, a key adaptation for waterlogging tolerant plants is the formation of adventitious roots (ARs). We found that cucumber waterlogging tolerant line Zaoer-N seedlings adapt to waterlogging stress by developing a larger number of ARs in hypocotyls, while almost no AR is generated in sensitive line Pepino. To understand the molecular mechanisms underlying AR emergence, the iTRAQ-based quantitative proteomics approach was employed to map the proteomes of hypocotyls cells of the Zaoer-N and Pepino under control and waterlogging conditions. A total of 5508 proteins were identified and 146 were differentially regulated proteins (DRPs), of which 47 and 56 DRPs were specific to tolerant and sensitive line, respectively. In the waterlogged Zaoer-N hypocotyls, DRPs related to alcohol dehydrogenases (ADH), 1-aminocyclopropane-1-carboxylicacid oxidases, peroxidases, 60S ribosomal proteins, GSDL esterases/lipases, histone deacetylases, and histone H5 and were strongly overrepresented to manage the energy crisis, promote ethylene release, minimize oxidative damage, mobilize storage lipids, and stimulate cell division, differentiation and growth. The evaluations of ethylene production, ADH activity, pyruvate decarboxylase (PDC) activity and ethanol production were in good agreement with the proteomic results. qRT-PCR analysis of the corresponding 146 genes further confirmed the accuracy of the observed protein abundance. These findings shed light on the mechanisms underlying waterlogging triggered cucumber ARs emergence, and provided valuable information for the breeding of cucumber with enhanced tolerance to waterlogging.

## Introduction

Waterlogging is described as the saturation of the soil with water around the plant roots, and constitutes one of the most severe abiotic stresses for plant growth and development (Sairam et al., 2009). Nearly 16% of the fertile areas of our globe annually undergo waterlogging due to excessive rainfall, lack of soil drainage, and irregular topography, which resulting in severe economic loss (Ahsan et al., 2007; Xu et al., 2014). In particular, the availability of oxygen for respiration is blocked in waterlogged organs because of gas diffusion in water is about 10^3^ times slower than that in the air (van Veen et al., 2014) To sustain energy supply, it is essential for the waterlogged organs to switch over to anaerobic mode for energy production (Jackson and Colmer, 2005; Xu et al., 2014). However, it is obviously inefficient to provide enough energy via glycolysis and fermentation when waterlogging is prolonged (Bailey-Serres and Voesenek, 2008; van Veen et al., 2014). To survive from long-term waterlogging stress, aerobic respiration for plants must be maintained via oxygen transport to enhance internal oxygen diffusion (Shimamura et al., 2014). Some species adapted to waterlogging stress by faster stem/hypocotyls elongation that enable the shoot to regain contact with the open atmosphere, such as *Rumex* and rice (Evans, 2004; Jiang et al., 2010).

Another key adaptation to waterlogging is the formation of ARs, which minimize the distance for oxygen diffusion and improve gas diffusivity (Sauter, 2013). ARs usually originated from the waterlogged part of hypocotyls or basal stem region, and such adaptation can replace the deteriorating primary roots (Bailey-Serres et al., 2012; Sauter, 2013) Therefore, the adaptive responses of ARs formation to waterlogging might be more important than those of primary roots for survival in waterlogged soil (Li et al., 2009; Yamauchi et al., 2014). Genetic, molecular, and physiological approaches have confirmed that auxin, ethylene and carbohydrate status can affect ARs formation (Changdee et al., 2009). A transgenic Arabidopsis line overexpressing the *auxin response factor* (*ARF*) *17* developed fewer ARs than wild-type plants, confirming the potential role of ARF genes in the regulation of ARs development by auxin (Sorin et al., 2005). Taramino et al. (2007) reported that the defective initiation of maize ARs in the *rootless concerning crown and seminal roots* mutant was caused by a mutation in the gene encoding the Lateral Oateral Boundaries (LOB) domain protein. Expression of the *LOB* gene was rapidly induced by the application of auxin, and the LOB protein is thought to be the direct targets of *ARF* transcription factor in the auxin signaling pathway. Rigal et al. (2012) have showed that overexpression of *AINTEGUMENTA LIKE 1 (PtAIL1)*, a transcription factor belongs to APETALA2/ETHYLENE RESPONSE FACTOR family, increased number of ARs in Populus, while RNA interference lines with a reduced level of *PtAIL1* transcripts had fewer ARs. However, the underlying mechanisms by which ARs formation is affected have not fully understood, and the reported findings have been contradictory. For examples, in flood-induced ARs of rice stem nodes, it is ethylene but not auxin that signals activation of the cell cycle (Lorbiecke and Sauter, 1999), which is followed by generation of ROS as measured with electron paramagnetic resonance spectroscopy (Voesenek and Bailey-Serres, 2015). Vidoz et al. (2010) found that there may be interaction between ethylene and auxin with respect to AR production in flooded tomato hypocotyls using ethylene- and auxin-insensitive mutants. These contradictory findings may be due to variation in the different plant species, growth conditions, and methods of quantifying ARs.

Cucumber is an economically important vegetable crop and is widely grown in the world with total harvest of more than two million hectares in 2016, ranking 4th in quantity of world vegetable production (FAO STAT 2016, http://faostat3.fao.org). Cucumber is also known for its sensitivity to waterlogging and easily affected by heavy rain or soil waterlogging due to its shallow root system and strict oxygen requirement (Qi et al., 2012; Xu et al., 2014, 2016). The relative tolerance of different cucumber accessions to waterlogging stress has been evaluated in our previous study (Qi et al., 2011). In greenhouse waterlogging experiment, we found that the waterlogging tolerant line Zaoer-N adapt to waterlogging treatment by developing numerous ARs in hypocotyls, while almost no ARs were generated in the sensitive line Pepino. As we known, proteomic applications provide a powerful tool for understanding plant proteome changes in response to abiotic and biotic stress (Chen and Harmon, 2006; Yang et al., 2013; Cui et al., 2015). In recent years, the iTRAQ-based quantitative proteomic approach has been widely used for the comparative analyses of proteome changes because it allows for the simultaneous identification and quantification of peptides by measuring the peak intensities of reporter ions with MS/MS (Yu et al., 2015). In the present study, an iTRAQ-based quantitative proteomics approach was employed to elucidate the detail effects of waterlogging stress on the protein expression levels in hypocotyls of the two contrasting cucumber lines differing in waterlogging tolerance ability and the capacity of ARs formation. The results will provide new insights into the physiological and molecular mechanisms associated with waterlogging stress in hypocotyls cells of cucumber seedling. To the best of knowledge, this is the first frame work that reveals the molecular mechanisms underlying AR emergence upon waterlogging on cucumber lines. Our findings provide a list of potential candidates for further elucidating waterlogging tolerance in plants.

## Materials and methods

### Plant growth and treatments

Waterlogging tolerant line Zaoer-N and sensitive line Pepino were grown in 8-cm-wide pots containing peat, vermiculite, and perlite (3:1:1, v/v/v) in a greenhouse at 28/20°C (14/10 h) day/night temperature and a relative humidity ranging from 70 to 85%. Cucumber seedlings at the three-leaf stage (21 days after germination) were placed in plastic cups filled with water (pH 7.03, 25°C, electrical conductivity 0.34 dS m^−1^, dissolved oxygen level 7.17 mg/L) to the base of the first true leaves (~4 cm above the soil surface, soil redox potentials Eh 272 ± 4.5 mV). Water was maintained at this level for the duration of the treatment. For control treatments, plants were watered daily and allowed to drain freely. Hypocotyls (below the water surface) were collected from the Zaoer-N and Pepino seedlings after 2 days of waterlogging and control for physiological assay. Thirty seedling hypocotyls from the three replicate were pooled, immediately frozen in liquid nitrogen, and then stored at −80°C for iTRAQ analysis and RNA extraction.

### Protein extraction and iTRAQ analysis

Cucumber hypocotyls were grinded to a fine powder in liquid nitrogen and then transferred to 700 μl extraction solution (8 M urea, 4% CHAPS, 2 M thiourea, 40 mM Tris/HCl, pH 8.5, 2 mM EDTA, 1 mM PMSF, 10 mM dithiothreitol). The mix was sonicated for 15 min, and then at 4°C centrifuged 25,000 g for 20 min. The supernatants were removed to a new tube and mixed well with 5 volumes of pre-chilled acetone incubated at −20°C for 2 h. After centrifugation, the resulting pellet was washed three times with cold acetone. The air-dried pellet was dissolved in the 250 μl of 0.5 M TEAB (Applied Biosystems, Milan, Italy), and sonicated for 15 min. The centrifugation step was repeated, and the supernatant was transferred to a new tube and protein concentration was quantified by the 2-D Quant kit (GE Healthcare) according to the manufacturer's protocol.

Hundred microliter of proteins for each sample were incubated with trypsin (Promega, Madison, WI, USA) at a ratio of protein: Trypsin = 20:1 overnight at 37°C. After digestion, the peptides were dried by vacuum centrifugation, reconstituted in 0.5 M TEAB. Labeling was performed according to the manufacturer's manual for the iTRAQ (Applied Biosystems, Foster City, CA USA) (113 tags for Zaoer-N control, 114 tags for Zaoer-N waterlogging treatment, 117 tags for Pepino control, 118 tags for Pepino waterlogging treatment). One unit of iTRAQ reagent (defined as the amount of reagent required to label 100 μg of protein) was thawed and reconstituted in 70 μL isopropanol. The labeled peptide mixtures were incubated for 2 h at room temperature and then dried by vacuum centrifugation. Peptides were purified using an SCX chromatography (LC-20AB HPLC pump system, Shimadzu Corporation, Kyoto, Japan). The fractions were then loaded into a Shimadzu LC-20AD nano HPLC by the auto sampler onto a 2 cm C18 trap column. Peptides were eluted at a flow rate of 300 μL/min with a linear gradient of 5–30% buffer B (95% ACN, 0.1% formic acid) over 60 min followed by ramping up to 100% buffer B over 5 min and holding for 20 min. The collected peptides were subjected to 5600 TripleTOFAnalyzer (AB Sciex).

The charge states of peptides were set to +2, +3, and +4. The mass spectrometer was maintained in positive ion mode, and MS spectra were acquired over a range of 300–1800 m/z. The resolving powers of the MS/MS and MS scan at 200 m/z for the Q-Exactive were set as 17,500 and 70,000, respectively. For the acquired MS spectra, the top ten most intense signals were collected for MS/MS analysis. Ions were fragmented through higher energy collisional dissociation with normalized collision energies of 30 eV, and the isolation window was 2 m/z. The maximum ion injection times were set at 10 ms for the survey scan and 60 ms for the MS/MS scans. The automatic gain control target values for both scan modes were set to 3.0 × 10^6^. The dynamic exclusion duration was 25 s. The chromatograms were recorded at 214 nm. The underfill ratio was defined as 0.1% on the Q-Exactive.

### Peptide and protein identification and proteomic data analysis

The raw mass files were analyzed by the Proteome Discoverer 1.4 software (Thermo Fisher Scientific). Search for the fragmentation spectra was performed by the MASCOT 2.3.02 software search engine embedded in Proteome Discoverer against the cucumber 9930 reference genome database version 2 (http://www.icugi.org/cgi-bin/ICuGI/index.cgi). The following search parameters were used: The initial precursor mass tolerance was set to ±20 ppm, and the fragment ion mass tolerance was set to ±20 mmu, trypsin as the cleavage enzyme. The protein identification was calculated by at least two unique peptides, minimum peptides for protein quantification was set to one, the normalization method and protein change ratio type (upregulated or downregulated) were both set as median. The results were filtered based on a false discovery rate (FDR) of no more than 1% to guarantee the result's confidence and a Mascot probability of 95%.

### Total RNA extraction and qRT-PCR

Total RNA from each sample was isolated by RNAiso Plus (Takara, China), then dissolved in water-DEPC and kept at a final concentration of 1000 μg/mL using Biophotometer Plus (Expander, Germany). Total RNA was reverse-transcribed by a Takara PrimeScript® RT reagent kit with gDNA eraser according to the manufacturer's manual (Takara, China). Primer sequences for qRT-PCR were designed using Primer Premier 5.0 (Premier Biosoft International, Palo Alto, CA, USA). qRT-PCR was performed using a Takara SYBR® PrimeScript™ RT-PCR kit (Takara, China). SYBR Green PCR cycling was performed on an iQ™ 5 Multicolor real-time PCR detection system (Bio-RAD, USA) using 20 μL samples with the following temperature program: 95°C for 10 s, followed by 40 cycles of 95°C for 15 s, and then annealing at 52°C for 30 s. The relative quantization of gene expressions were calculated and normalized to *Actin*. Three replicates were used for qRT-PCR.

### Physiological determination

The chlorophyll concentration was measured by a SPAD (soil and plant analysis development) meter (SPAD-502Plus, Konica Minolta, Tokyo, Japan). 7 days after removing waterlogging treatment, cucumber plants were visually compared with well-watered control and scored for tolerance rating (TOR) using a scale of 0–5 (Navazio and Staub, 1994), where 0 = dead plants, 1 = 75 ~100% of wilt, 2 = 50~74% wilt, 3 = leaves undulating and recurved, 4 = recurved leave margins and 5 = green plant with no sign of stress. The lower scale stood for waterlogging susceptibility while the higher scale stood for tolerance. Ethylene production after 48 h of waterlogging treatment was measured by a gas chromatograph device equipped with a TRB-5 capillary column (0.32 mm id, 30 m length, 0.25 lm df) and a flame ionization detector. Temperature, injection, detector and heat clamber 150, 150, and 100°C were set. Nitrogen was employed as the carrier gas. PDC (EC 4.1.1.1) and ADH (EC 1.1.1.1) activities were detected by spectrophotometer (Spectrum SP-752, Shanghai, China) metrically at 340 nm, following the methods described by Xu et al. (2014). Crude enzymes extracts of hypocotyls were prepared by macerating 1.0 g of the tissue in 5.0 cm^3^ of 0.1 M cold Tris-HCl buffer (pH 8.0) as described by Mustroph and Albrecht (2003). ADH and PDC activity was determined spectrophotometrically by measuring the rate of reduction of NAD^+^ (Waters et al., 1991). The reaction mixture contained 30 mM ethanol, 65 mM Tris-HCl buffer (pH 9.5), 1 mM NAD+, and 0.1 cm^3^ of crude enzyme extracts. Increase in absorbance due to production of NADH was recorded at room temperature. 1 U of PDC and ADH was defined as the amount of enzyme required to decompose 1 mol of substrate per g protein per minute. For ethanol production experiment, fresh hypocotyls were harvest and frozen rapidly in liquid nitrogen and ground to a powder. 500 mg root material was homogenized with 50 Mm cold 0.1 M HCl in a Teflon-sealed test tube. After 20 min incubation at 70°C, a 1 mL sample of headspace was injected into a gas chromatograph (Thermo Trace GC Ultra) equipped with a supel cowax 10 column (30 m × 0.32 mm × 0.25 μm) and a flame ionization detector following the instructions of Kato-Noguchi and Watada (1997). Ethanol evaporation under these conditions was negligible.

## Results

### Phenotype differences of Zaoer-N and pepino in responding to waterlogging treatment

To evaluate the phenotype differences of waterlogging stress on the tolerant Zaoer-N and the sensitive Pepino, seven representative phenotypes after 7 days of waterlogging were recorded. As is shown in Table 1, the tolerance rating, SPAD value, leaf flesh weight, ARs number, and flesh weight for Zaoer-N were considerably greater than those for Pepino, indicating that Zaoer-N was significantly more tolerant than Pepino (*p* < 0.05). However, the differences between Zaoer-N and Pepino for primary root flesh weight and hypocotyls length were not significant which suggesting the primary root injury and hypocotyls elongation may not contribute to waterlogging tolerance between the two lines.

**Table 1:** **Response of waterlogging tolerant line Zaoer-N and sensitive line Pepino after 7 days of waterlogging treatment**.

**Trait**	**Zaoer-N control**	**Zaoer-N waterlogged**	**Pepino control**	**Pepino waterlogged**
TOR	5 ± 0a	4.67 ± 0.33a	5 ± 0a	1.09 ± 0.16b
SPAD	41.89 ± 1.24a	39.71 ± 2.41a	43.03 ± 0.56a	21.50 ± 1.34b
ARN	0 ± 0 bc	29.43 ± 2.5a	0 ± 0 bc	2.17 ± 1.43b
HLH	5.12 ± 0.27a	5.23 ± 0.22a	5.26 ± 0.77a	5.35 ± 0.67a
LFW	0.48 ± 0.14a	0.36 ± 0.09a	0.56 ± 0.17a	0.19 ± 0.1ab
HFW	0.92 ± 0.11b	1.6 ± 0.36a	0.8 ± 0.03a	0.4 ± 0.23b
PRFW	0.36 ± 0.05a	0.13 ± 0.11b	0.42 ± 0.1a	0.12 ± 0.07b

*TOR, tolerance rating = 0 (dead plants)—5 (green plants with no sign of symptom); SPAD, soil plant analysis development; ARN, adventitious root numbers; HLH, hypocotyls length; LFW, leaf flesh weight; HFW, hypocotyls flesh weight; PRFW, primary root flesh weight. Means within trait followed by the same letter(s) are not significant difference at p-value < 0.05*.

**Figure 1:**
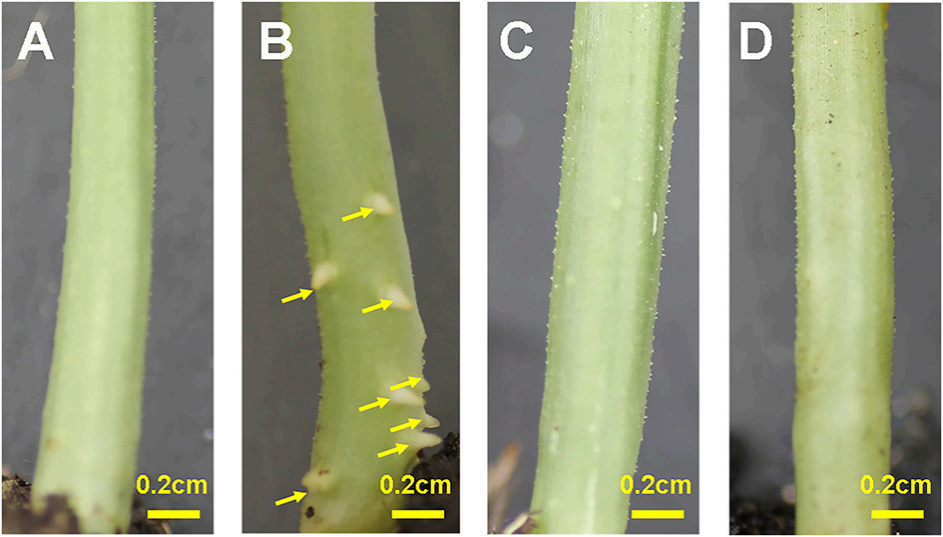
**Adventitious root formation on hypocotyls of cucumber seedlings**. **(A)** Zaoer-N control; **(B)** Zaoer-N waterlogging; **(C)** Pepino control; **(D)** Pepino waterlogging. The yellow arrows in the picture indicated the location of adventitious roots. The water level was kept at about 2 cm above the soil for 2 days and then removed for photography.

Transferring Zaoer-N seedlings to waterlogging stress resulted in ARs primordia emergence and visible on the hypocotyls surface 2 days after waterlogging, in comparing with control plants and waterlogged Pepino (Figure 1). Seven days after treatment, the average AR numbers protruded at the basal part of the hypocotyls were 30.4 (Figure S1). To elucidate the response mechanisms related to morphological differences between the two genotypes in responding to waterlogging, an iTRAQ proteomic approach was thus applied to the hypocotyls of the two-day-waterlogged Zaoer-N and Pepino seedlings.

### Inventory of hypocotyls proteins identified by iTRAQ

**Figure 2:**
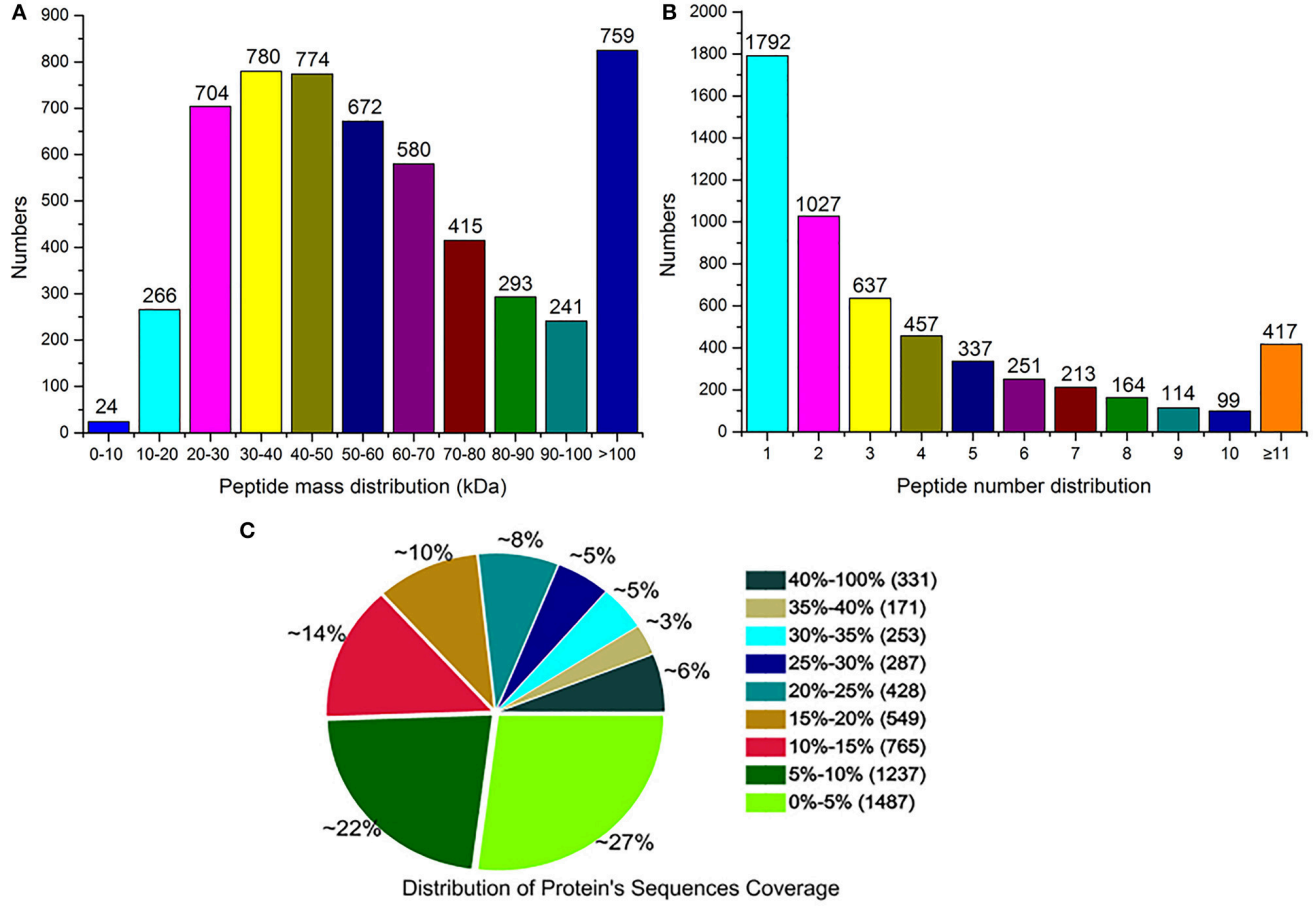
**Basic information of iTRAQ output**. **(A)** distribution of the proteins that were identified among different molecular weights. **(B)** number of peptides that match proteins using MASCOT. **(C)** coverage of the proteins by the identified peptides.

Using the Mascot software, a total of 52,053 spectra matched to known spectra, 46,773 spectra matched to unique peptides, and 23,533 peptides, 22,066 unique peptides, and 5508 proteins were identified (Table 2). All the mass spectrometry data have been deposited into the ProteomeXchange Consortium via the PRIDE partner repository with the dataset identifier PXD004023. Among these proteins, 1048 were between 20 to 60 kDa, 129 between 0 to 20 kDa, 379 between 60 to 100 kDa, and 175 over 100 kDa (Figure 2A). 1792 proteins had one identified peptide, 1027 had two, 417 had more than 11, and the left had 3–10 (Figure 2B). The peptide sequence coverage was primarily less than 20% (Figure 2C). Ratio distributions for the identified proteins in Zaoer-N and D8 were shown in Figures S2A,B, respectively. Because iTRAQ quantification underestimated the amount of real fold change between two samples, protein with a fold-change >2 or < 0.5 in abundance were regarded as DRPs. Based on this criteria, 146 DRPs in the Zaoer-N and/or Pepino were selected for further analysis. Of these, 90 DRPs (62 increased and 28 decreased in abundance) changed significantly in the tolerant Zaoer-N and 99 DRPs (62 increased and 37 decreased in abundance) in the sensitive Pepino (Table S1). Only 43 DRPs (34 increased and 9 decreased in abundance) were shared between the two lines, while 47 DRPs were only identified in Zaoer-N, and 56 DRPs were only identified in Pepino.

**Table 2:** **Spectra, peptides, and proteins that were identified from iTRAQ proteomics by searching against the cucumber reference genome 9930 version 2 database**.

**#**	**Category**	**Number**
1	Total spectra	331,676
2	Spectra	52,053
3	Unique spectra	46,773
4	Peptide	23,533
5	Unique peptide	22,066
6	Protein	5508

### Classification of waterlogging responsive DRPs

**Figure 3:**
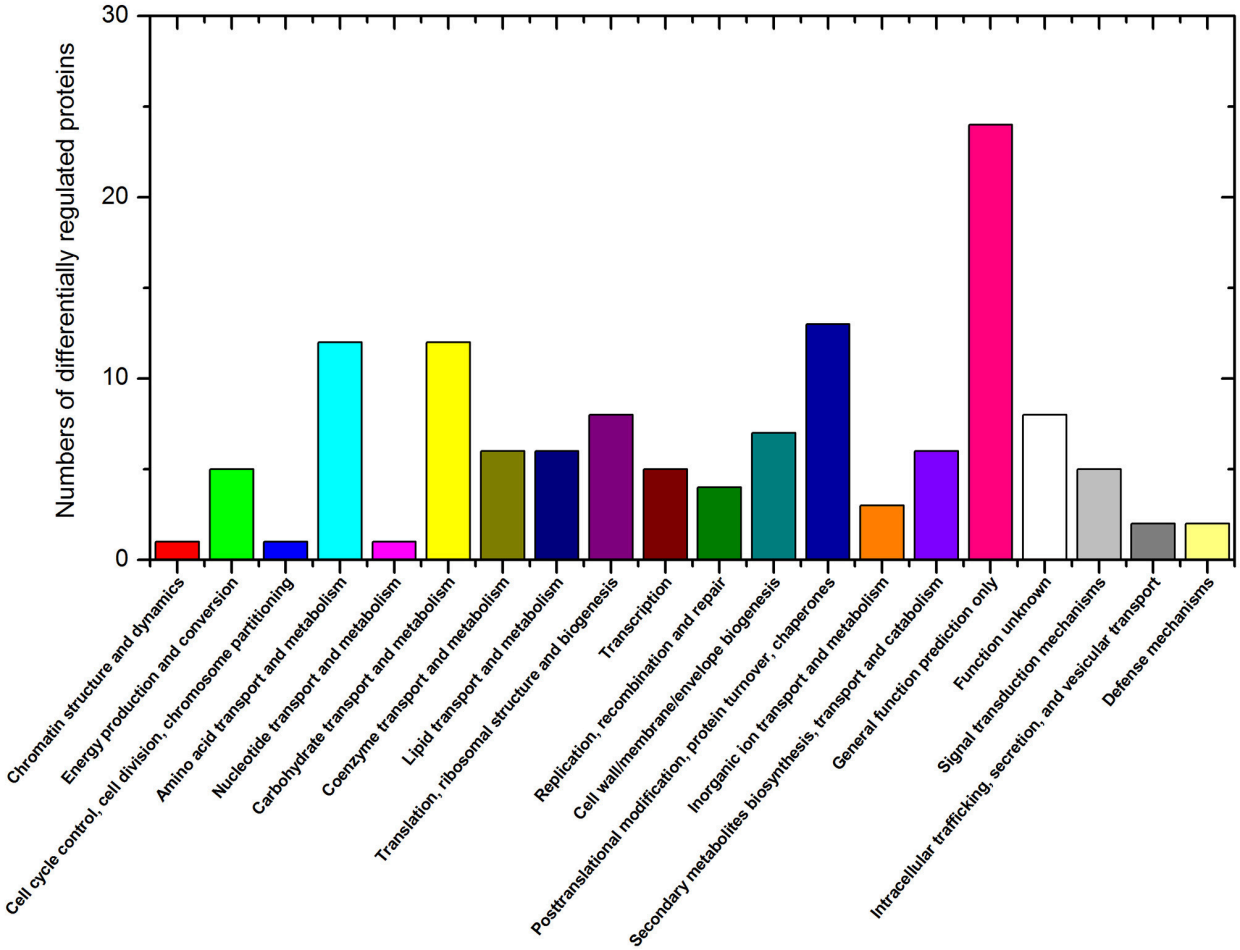
**Clusters of Orthologous Groups of proteins (COG) classification of the differentially regulated proteins**.

**Figure 4:**
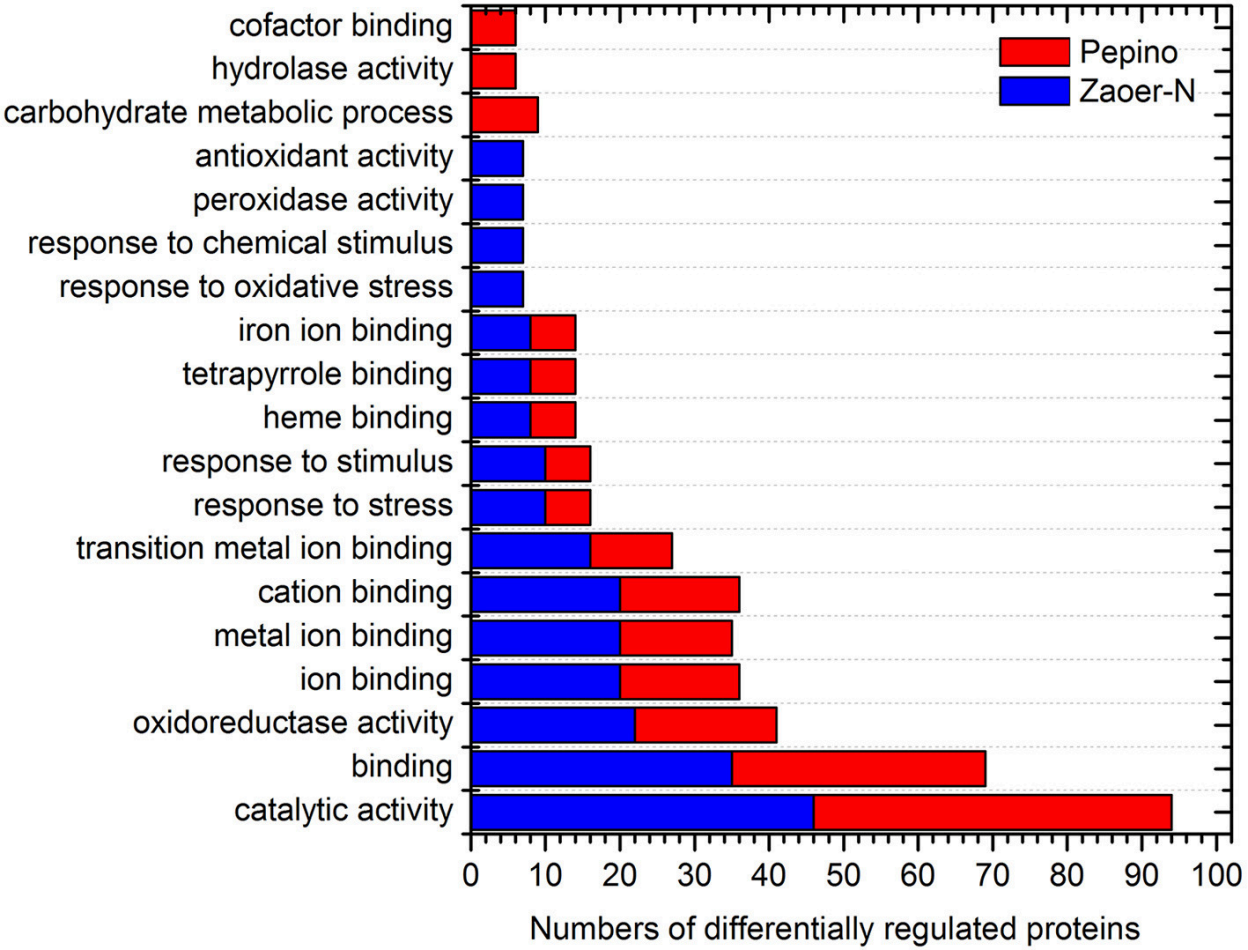
**Functional classification of differentially regulated proteins identified in this study**. AgriGO web-based program was used to analyze GO categories (http://bioinfo.cau.edu.cn/agriGO/). The X-axis is the numbers of differentially regulated proteins. The Y-axis is categories of GO term.

Of the 146 DRPs, 131 DRPs can be assigned to 21 categories using the COG database. The largest group was general function prediction only (18.3%) followed by posttranslational modification, protein turnover, chaperones (9.9%), carbohydrate transport and metabolism (9.2%), amino acid transport and metabolism (9.2%) and Translation, ribosomal structure and biogenesis (6.1%) (Figure 3). To further characterize these identified waterlogging responsive proteins, the DRPs identified in Zaoer-N and Pepino were respectively passed through GO enrichment analysis using the agriGO (http://bioinfo.cau.edu.cn/agriGO). A GO term was considered to be significantly enriched if the false discovery rate was below 0.05. Enrichment analysis of GO functions revealed that 12 GO terms were found shared between Zaoer-N and Pepino, such as oxidoreductase activity (GO:0016491), response to stress (GO:0006950) and heme binding (GO:0020037). Four GO terms were unique to Zaoer-N, including response to oxidative stress (GO:0006979), response to chemical stimulus (GO:0042221), peroxidase activity (GO:0004601), and antioxidant activity (GO:0016209). On the contrary, GO terms related to carbohydrate metabolic process (GO:0005975), hydrolase activity (GO:0016798), and cofactor binding (GO:0048037) were specially enriched in Pepino (Figure 4).

To characterize the functional consequences of the DRPs associate with waterlogging stress and subsequent AR formation, KEEG pathway mapping based on KEGG orthology terms for assignment was also carried out. Only significantly enriched pathway categories that had a *p-value* lower than 0.05 were selected. The results indicated that waterlogging could affect phenylpropanoid biosynthesis and alpha-Linolenic acid metabolism in both of the two lines. KEEG terms “glycolysis/gluconeogenesis” and “biosynthesis of secondary metabolites” were the highly enriched in the DRPs that were specifically up-regulated in the Zaoer-N, but “arginine biosynthesis,” “alanine, aspartate, and glutamate metabolism,”and “biosynthesis of amino acids” were dramatically enriched in DRPs in Pepino (Table 3). All of the DRPs in KEGG categories were shown in Tables S2, S3.

**Table 3:** **KEGG pathway enrichment analysis of differentially regulated proteins (DRPs)**.

**#**	**KEEG term**	**Pathway ID**	**DRPs in Zaoer-N**	***P*-value**	**DRPs in Pepino**	***P*-value**
1	Phenylpropanoid biosynthesis	csv00940	9	2.94 E-04	7	0.005
2	alpha-Linolenic acid metabolism	csv00592	4	0.005	3	0.031
3	Biosynthesis of secondary metabolites	csv01110	20	0.0176	0	–
4	Glycolysis / Gluconeogenesis	csv00010	5	0.016	0	–
5	Biosynthesis of amino acids	csv01230	0	–	6	0.046
6	Arginine biosynthesis	csv00220	0	–	3	0.012
7	Alanine, aspartate and glutamate metabolism	csv00250	0	–	3	0.028

*KOBAS2.0 web-based program was used for KEEG mapping (http://kobas.cbi.pku.edu.cn/)*.

### Protein–protein interaction analysis

**Figure 5:**
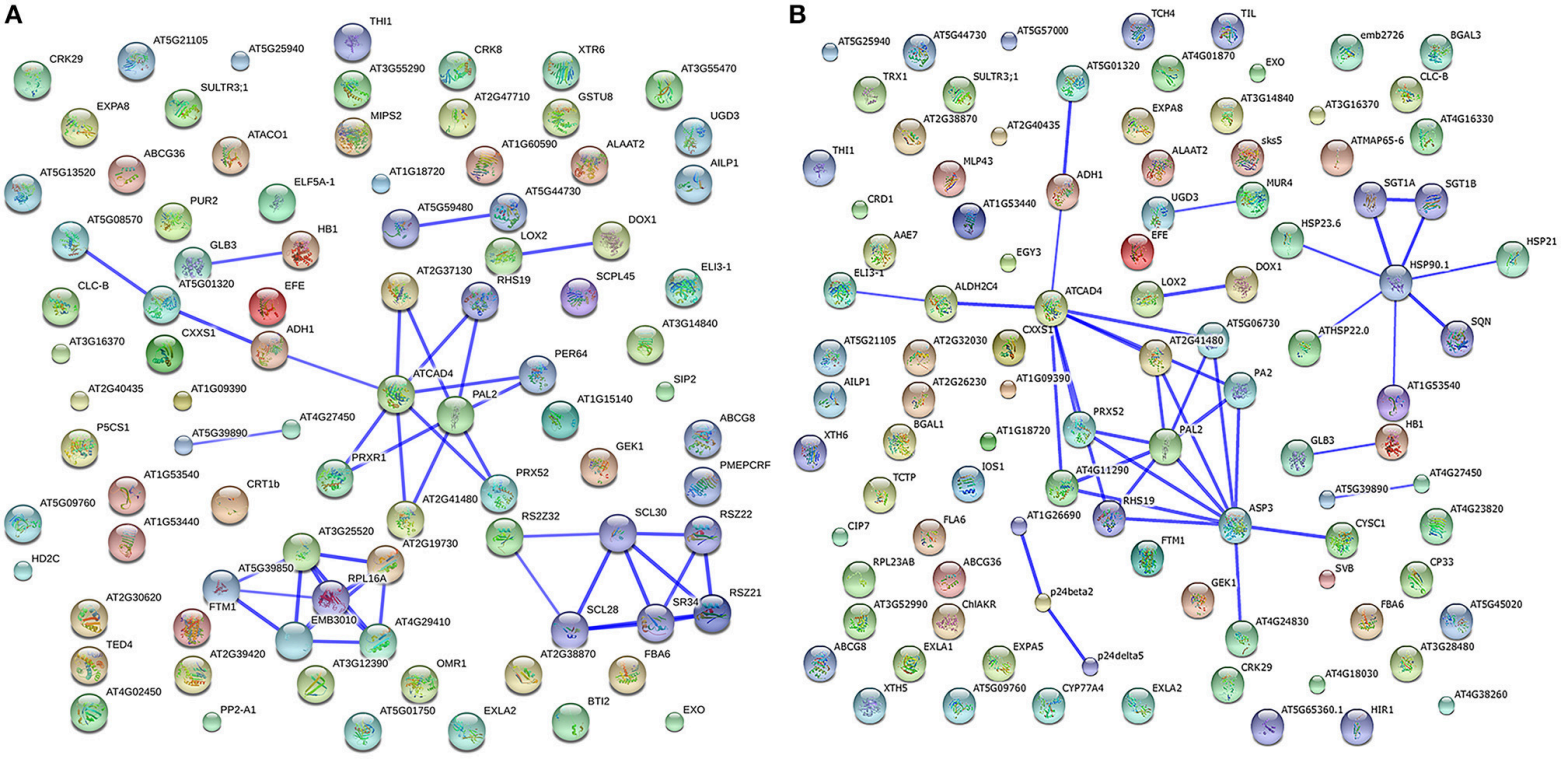
**Interaction network analysis of differentially regulated proteins**. The interaction network as analyzed by STRING software (http://string-db.org). In this network, nodes are proteins; lines represent functional associations between proteins. The resulting networks were constructed with confidence scores higher than 0.7. The blue lines between bodes represent functional associations between proteins and the thickness of the lines represents the level of confidence in association reported. **(A)** network analyzed from waterlogging responsive proteins in Zaoer-N hypocotyls. **(B)** network analyzed from waterlogging responsive proteins in Pepino hypocotyls.

Plant proteins play interrelated roles together in the context of networks. To investigate how the cucumber hypocotyls transmit waterlogging stress signaling through protein–protein interactions, the identified DRPs in Zaoer-N and Pepino were analyzed respectively using the String database (http://string-db.org). The abbreviations of the protein names in the networks were shown in Table S4. Eight separate interaction networks were predicted in Zaoer-N (Figure 5A). The ribosomal proteins, including 40S ribosomal protein (AT5G39850), 60S ribosomal proteins (RPL16A, AT5G02870, AT1G57660, AT2G19730, AT4G29410), ribosomal protein L5 (AT3G25520), were found to be actively interacted with embryo defective 3031 (EMB3031). Another notable interacted protein group is composed of pyruvate kinase (AT5G08570), PDC (AT5G01320), ADH1, cinnamyl alcohol dehydrogenase 1 (ATCAD4), Prxs (PRXR1, AT2G41480, PRX52, PER64, RHS19, and AT2G37130), and phenylalanine ammonia-lyase 2 (PAL2). Argine/serine-rich zine knuckle-containing protein 33 (RS2Z33) was a key protein in a another network and interacted with pre-mRNA-splicing factor SR34, serine/arginine-rich protein RSZ22, zine finger protein RSZ21, SC35-like splicing factors (SCL30A, SCL30, and SCL28). Furthermore, there were five pairs of interactive protein-species in this network. The main two pairs were hemoglobin 3 (GLB3) and hemoglobin 1 (HB1), deoxyhypusine synthase (DHS) and eukaryotic elongation factor (ELF5A-1), lipoxygenase 2 (LOX2) and alpha-dioxygenase (DOX1), haloacid dehalogenase-like hydrolase domain-containing protein (AT5G59480) and haloacid dehalogenase-like hydrolase domain-containing protein (AT5G44730), uncharacterized protein (AT5G39890) and aluminum induced protein (AT4G27450), respectively. Seven separate interaction networks were predicted in Pepino, and some of them were overlapped with the prediction in Zaoer-N (Figure 5B). However, the network made up of heat shock proteins (HSP90.1, HSP23.6, ATHSP22.0, AT1G53540, and HSP21), phosphatases (SGT1A and SGT1B) and SQUINT were only found in Pepino. Although these predicted interaction networks need to be verified, they have provided a narrow pool of protein–protein interactions in cucumber hypocotyls in responding to waterlogging for our further investigations.

### Correlation of iTRAQ data with mRNA expression data

**Figure 6:**
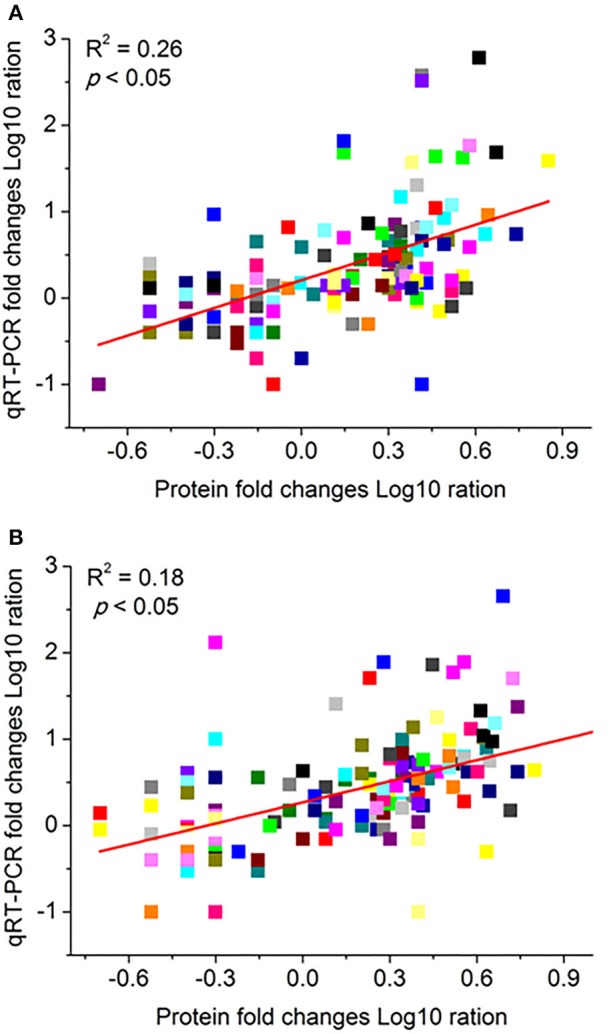
**Comparison of protein folds change levels measured by iTRAQ and quantitative real-time reverse transcription-PCR (qRT-PCR) assays in Zaoer-N (A) and Pepino (B)**. The gene expression values were transformed to the log10 scale. The protein fold changes log10-values (X-axis) were plotted against the qRT-PCR fold changes log2-values (Y-axis). The cucumber β -actin gene (GenBank AB010922) was used as an internal control to normalize the expression data. Each value denotes the mean relative level of expression of three biological replicates.

The correlation of iTRAQ data and mRNA expressions were carried out to further investigate protein expression profiles under waterlogging stress. The mRNA expressions levels were obtained by qRT-PCR analysis of the corresponding 146 genes with the same experimental design. In the correlation analysis, we calculated the Pearson's correlation coefficients (PCC-value). The PCC-values of the DRPs and mRNA pairs were 0.26 in Zaoer-N (Figure 6A), while PCC-values were 0.18 for groups in Pepino, respectively (Figure 6B). As expected, significant correlations existed in both of the two groups at the 0.05 level (2-tailed). The positive correlation of iTRAQ data and mRNA expression levels suggested that the regulation of these proteins likely resulted from the transcriptional inductions of the corresponding genes in the waterlogged hypocotyls cells. However, the results also showed that some proteins and corresponding gene relative expression levels were not well correlated. The discrepancy of these protein and mRNA expression levels also has been found in other iTRAQ studies, and may be ascribed to translational and posttranslational regulatory processes or feedback loops between the processes of mRNA translation and protein degradation(Vogel and Marcotte, 2012).

### Physiological responses of Zaoer-N and pepino to waterlogging

**Figure 7:**
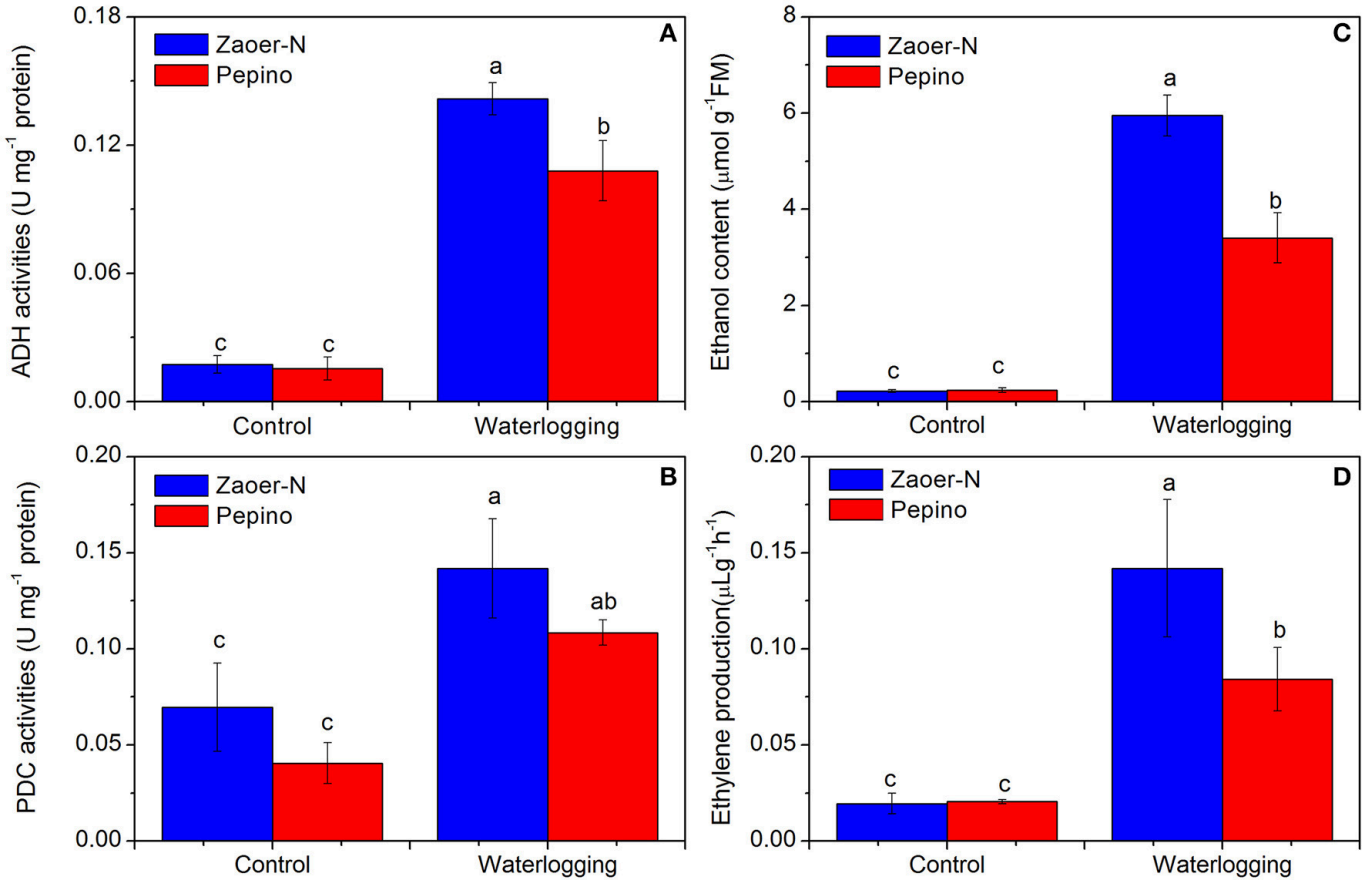
**Physiological responses of Zaoer-N and Pepino hypocotyls to waterlogging stress**. Hypocotyls of control and treated plants were harvested as described in Materials and methods. Four physiological parameters, including alcohol dehydrogenase (ADH) activity **(A)**, pyruvate decarboxylase (PDC) activity **(B)**, ethanol production **(C)**, and ethylene production **(D)** were measured. Data represents the average values ± SE of three independent experiments. Letters above the bars indicate a statistically significant difference (*p* < 0.05) according to Duncan's multiple range test.

To investigate whether these identified DRPs can result in physiological changes, we also evaluated the ethylene release, ADH and PDC activities and production of ethanol (Figure 7). Two days of waterlogging treatment led to a significant increase in ADH and PDC activities (*p* < 0.05) and both of them were higher in Zaoer-N than that in Pepino (Figures 7A,B). As we known, the production of ethanol closely correlated with ADH activity. Compared with non-waterlogged cucumber hypocotyls, the ethanol concentrations after 2 days of waterlogging increased significantly (*p* < 0.05) in both lines but to a greater degree in Zaoer-N than in Pepino (Figure 7C). A 7.3-fold and 4.1-fold increase in ethylene release after 2 days of waterlogging was observed in hypocotyls of Zaoer-N and Pepino, respectively (Figure 7D).

## Discussion

ARs formation as a result of flooding or waterlogging stress were reported for tomato (Vidoz et al., 2010), bittersweet (Dawood et al., 2014, 2016), wheat (Barrett-Lennard et al., 1988), barley (Pang et al., 2007), rice (Bleecker et al., 1986), and maize (Mano et al., 2005). In deep-water rice cultivar *Pin Gaew56*, AR primoridia emerged from the stem node within 8 ~ 10 h of partial submergence (Lorbiecke and Sauter, 1999). The present study showed that ARs primordia emergence and visible on the cucumber hypocotyls surface 2 days after waterlogging, the number of ARs significantly increased in Zaoer-N compared with Pepino 7 days after waterlogging, and the SPAD value in the leaves was significantly higher in Zaoer-N than in Pepino (Table 1). These observations indicated that Zaoer-N adapted better to waterlogging stress than Pepino. ARs facilitate oxygen diffusion in Zaoer-N to accelerate aerobic metabolism, and maintaining a sufficient energy supply to meet the demands of the long-term survival (Bailey-Serres and Voesenek, 2008). The process of activation of AR emergence under waterlogging, however, has been studied to a much lesser extent. To further our understanding about the molecular and physiological reprogramming that enable AR primordia to resume growth upon an abiotic trigger, we thus analyzed the proteomic response to waterlogging in cucumber at the initial stage of ARs growth.

### Management of the energy crisis

Energy crisis is a major factor affecting waterlogged organs survival because only 2 ~ 4 mol ATP per mol hexose were produced by glycolysis and fermentation when compared with 30 ~ 36 mol ATP produced by aerobic respiration (Bailey-Serres and Voesenek, 2008). Previous studies have confirmed that both sensitive and tolerant plants are able to maintain their ATP production by inducing glycolytic and fermentative enzymes at the early adaptive response to waterlogging treatment (Yu et al., 2015). As expected, we also found that several enzymes, such as UDP-glucose 6-dehydrogenase (Csa1M012150.1) in hemicellulose synthesis, fructose-bisphosphate aldolase (Csa3M750920.1), and pyruvate kinase (Csa5M580610.1 and Csa6M449830.1) involved in glycolysis, and ADH (Csa7M320050.1) and PDC (Csa6M518930.1) in ethanol fermentation were significantly accumulated in both of Zaoer-N and Pepino, suggesting the common regulation of energy generation in waterlogged cucumber hypocotyls. However, our data showed that ADH (Csa7M322060.1) accumulated only in Zaoer-N (Table 4, Table S1), in accordance with the observation of ADH activity and ethanol concentrations (Figures 7A,C). As we known, ADH plays an essential role in the recycling of NADH to NAD^+^, which is pivotal for the continuation of glycolysis pathway (Ismond et al., 2013), and is the only energy source under oxygen deprivation condition. Interestingly, aldehyde dehydrogenase (ALDH, Csa1M372010.1), which converts the acetaldehyde into acetate, was accumulated only in Pepino. As postulated by Nakazono et al. (2000), the conversion of acetaldehyde to acetate by ALDH also consumes NAD^+^, which could potentially further block glycolysis and ATP supply. Together, these results suggested that the two lines exhibit a similar enzymes cascade as other plants species in responding to waterlogging stress, but the more efficient of regenerate ATP and NAD^+^ and continuation of the glycolysis pathway in tolerant Zaoer-N than sensitive Pepino to cope with energy crises imposed by waterlogging. Nevertheless, high rates of glycolysis and alcohol fermentation will unavoidably lead to the fast depletion of sugar stores and carbon crisis, and the products of alcohol fermentation (acetaldehyde and ethanol) may have damaging consequences on cell integrity (Limami et al., 2008). Thus, escape from the damaging consequences appears an important issue in survival strategy, especially for those tolerant lines if waterlogging stress prolonged.

**Table 4:** **Information of differentially regulated proteins related to alcohol dehydrogenase, pyruvate decarboxylase, and 1-aminocyclopropane-1-carboxylate oxidase**.

**Protein name**	**Function**	**Coverage (%)**	**No. of unique peptides**	**Fold changes (waterlogging vs. controls)**	
				**Zaoer-N**	**Pepino**
Csa7M320050.1	alcohol dehydrogenase	49.2	9	3.333	4.484
Csa7M322060.1	alcohol dehydrogenase	60.7	11	3.788	–
Csa6M518930.1	pyruvate decarboxylase	22.8	7	2.628	2.075
Csa2M000520.1	1-aminocyclopropane-1-carboxylate oxidase	27	7	4.651	–
Csa4M056660.1	1-aminocyclopropane-1-carboxylate oxidase	8.8	3	2.050	2.062
Csa6M511860.1	1-aminocyclopropane-1-carboxylate oxidase	27.2	5	2.500	2.32

### Ethylene production and ROS production in responses to waterlogging

Among several internal changes in the waterlogged plants, it is the pervasive and rapid accumulation of ethylene that makes it an early and reliable waterlogging-triggered signal (Sasidharan and Voesenek, 2015). In our previous study, we found that the ARN in Zaoer-N hypocotyls 3 days after waterlogging was significantly inhibited when pretreated with 100 mg/ L 1-methylcyclopropene as an inhibitor of ethylene action (Xu et al., 2016), suggesting the importance of ethylene in waterlogging-triggered cucumber AR production. Ethylene production was observed in both of Zaoer-N and Pepino 2 days after treatment (Figure 7D). As is known to all, the gaseous phytohormone ethylene is biosynthesized from methionine, and it is produced by the activation of 1-aminocyclopropane-1-carboxylicacid (ACC) synthase and ACC oxidase (ACO) (Yang and Oetiker, 1994). We identified three ACOs (Csa2M000520.1, Csa4M056660.1, and Csa6M511860.1), and two of these proteins were accumulated in both lines, whereas Csa2M000520.1 accumulated only in Zaoer-N, indicating that a higher quantity of ethylene production in waterlogged Zaoer-N than those in Pepino may caused by accumulation of this protein (Table 4, Table S1). It should be pointed out that S-adenosyl-L-methionine synthetase (SAM, Csa3M016410.1) was decreased following exposure to 2 days of treatment. SAM is the key enzyme in the synthesis of S-adenosyl-L-methionine, which is an important metabolic component in the ethylene biosynthesis pathway (Yang and Hofman, 1984). The decrease of SAM in the proteome from waterlogging-treated tomato root has also been reported (Ahsan et al., 2007). The decrease of SAM observed in the current study indicated that ethylene production in cucumber hypocotyls would be reduced after 2 days of treatment. In deep-water rice, the enhanced formation of epidermal cell death at sites where ARs primordia emerge is dependent on the presence of ethylene (Mergemann and Sauter, 2000; Steffens et al., 2011). The growing ARs exert a mechanical force on the epidermal cells overlying them in a process that also requires ethylene-mediated ROS generation (Steffens et al., 2006). Despite of ROS at lower concentrations may act as signaling molecules involved in acclimation to environmental stress, a high level of these compounds are also harm to the plant cell. Heat shock proteins (HSPs) are the most recurrent of ROS related proteins that not only respond to heat stress, but also in respond to other stress, such as waterlogging, and has been proven to be associated with the presence of hydrogen peroxide (Pucciariello et al., 2012; Banti et al., 2013). Five HSPs were identified in present study, and all of them (Csa3M113300.1, Csa3M020080.1, Csa3M183950.1, Csa5M138480.1, and Csa5M591720.1) were accumulated only in Pepinos. Increase of HSPs has been proposed to be specific H_2_O_2_ sensors in plants, suggesting the higher H_2_O_2_ production in Pepino.

### Proteins involved in cell division and cell growth

The process of AR primordia emergence is accompanied with extensive cell division and cell growth. Regulation of cell division and cell growth-related proteins were also apparent. The DRPs list contained 14 cell wall remolding proteins, including expansins, pectinesterase, proteinase inhibitor, xyloglucan endotransglucosylase/hydrolases, polygalacturonase, and some structural constituent of cell wall. Most of these proteins had higher fold changes in Zaoer-N than Pepino, suggesting the AR primordia emergence in Zaoer-N could not be separated with cell wall extensibility. Interestingly, nine peroxidase (Prxs), which involved in many development processes such as cell growth and differentiation (Dunand et al., 2007), were also differentially regulated. Five of them (Csa6M495000.1, Csa7M049140.1, Csa7M061710.1, Csa2M346020.1, and Csa3M736970.1) were accumulated only in the waterlogged hypocotyls of Zaoer-N. Passardi et al. (2006) found that Arabidopsis seedlings lacking *Prx33* transcripts have shorter roots than the wild-type controls and roots are still shorter in the double mutant, while seedlings overexpressing *AtPrx34* exhibit significantly longer roots. Studies have also provided evidences that Prxs activities play an protective role in the ROS scavenging process upon waterlogging stress (Qi et al., 2012). Thus, accumulation of Prxs in Zaoer-N will help consumption of ROS and promote cell elongation around the AR primordia. Additionly, our iTRAQ data also showed that two histone deacetylases (Csa3M748810.1 and Csa6M150540.1) were decreased in Zaoer-N specially, which might results in decondensation of the chromatin structure around the promoter regions of embryonic genes and help stimulating cell differentiation (Yoo et al., 2006). The Arabidopsis histone deacetylases HDA6 and HDA19 redundantly regulate embryonic genes and negatively affect callus formation from the hypocotyls (Tanaka et al., 2008). Thus, decrease of these histone deacetylases may also help promoting the emergence of AR primordia in Zaoer-N hypocotyls. Although we did not detect cyclin or cyclin dependent kinase in the hypocotyls of Zaoer-N at the 2 days time point, there were already a specific induction of many proteins controlling cell size and cell proliferation in Zaoer-N hypocotyls, such as 60S ribosomal proteins, which are absolutely required for protein synthesis (Bailey-Serres and Freeling, 1990), and histone H5 (Csa6M407650.1) in cell division (Bailey-Serres and Voesenek, 2008). In contrast to the situation in the primordia, a large group of cell wall modifying- and cell cycle-related proteins were decreased in the hypocotyls of Pepino. These findings suggest that cell division and growth are suppressed in Pepino hypocotyls upon waterlogging, such that these hypocotyls might enters a state of endurance.

### Proteins related to jasmonic acid (JA) content

The threonine dehydratase (TD, Csa6M448730.1) was the highest decreased (~0.048 fold than control) protein that specifically regulated in waterlogged Zaoer-N. TD catalyzes the formation of α-keto butyrate from Thr, the first step in the biosynthesis of Ile (Kang et al., 2006). For more than a decade, TD has been recognized as a reliable marker for JA elicitation in potato and tomato (Dammann et al., 1997). This information prompted us to investigate the JA concentration during AR emergence on cucumber hypocotyls. As expected, JA concentrations in 2-day waterlogged Zaoer-N hypocotyls were significantly decreased (~0.33 fold) than control. On the contrary, JA concentrations in Pepino hypocotyls were significantly increased (~1.99 fold) than control after 2 days of waterlogging (Table S5). Sanders et al. (2000) and von Malek et al. (2002) found that JA deficient mutants *opr3/dde1* and *dde2-2* produced more ARs than the wild type, Gutierrez et al. (2012) showed that Arabidopsis *gh3* triple mutant accumulated twice as much JA but inhibited the AR initiation compared with the wild type. It is tempting to speculate that JA is an inhibitor of cucumber adventitious rooting. The accumulation of JA in waterlogged Pepino might be caused by the higher fold changes of lipoxygenase (Csa4M286960.1, ~6.29 fold than control, Table S1), which is a key enzyme in JA biosynthesis. However, due to the oxygen requirement of lipoxygenase (Arbona and Gómez-Cadenas, 2008), the increased JA biosynthesis via lipoxygenase pathway will unavoidably lead to higher risk of being injured by oxygen deprivation under waterlogging condition.

### Proteins related to phenylpropanoid metabolism and lipid catabolism

A feature of plant responses to waterlogging is the activation of phenylpropanoid metabolism, which is also supported by our KEEG pathway mapping (Table 3). We found that DRPs enriched in KEEG term “phenylpropanoid metabolism” (Table S3) were all Prxs (as described in Proteins Involved in Cell Division and Cell Growth Section) except for a phenylalanine ammonia-lyase (PAL, 4.3.1.5). PAL catalyzes the first committed step of the core pathway of general phenylpropanoid metabolism (Gómez-Vásquez et al., 2004), and is one of the most extensively studied enzymes with respect to plant responses to abiotic stress. Higher levels of PAL activity or mRNA accumulation in response to waterlogging have been found in tomato roots, *Vigna sinensis* and *Zea mays* (Alla et al., 2002). However, in current investigation, we found that PAL (Csa6M147460.1) decreased in cucumber hypocotyls after 2 days of waterlogging treatment, and to a greater degree in Zaoer-N (0.071) than in Pepino (0.456). Politycka (1998) found that the upregulation of PAL activity resulted in cucumber root growth decreasing. The highly inhibition of PAL enzyme could result from accumulation of its intermediate product in phenylpropanoid metabolism i.e., the trans-cinnamic acid, and we thus speculated that the decrease of PAL in waterlogged Zaoer-N could benefit to waterlogging triggered AR growth.

In plants, lipid catabolism is known to be an active process that can mobilize storage lipids and can detoxify deleterious membrane lipids caused by abiotic stresses (Kim et al., 2016). Except for the lipoxygenase discussed above, our iTRAQ study also found that three GSDL esterases/lipases, Csa1M058140.1, Csa3M669600.1, and Csa3M434970.1, were differentially regulated by waterlogging stress. GDSL esterases/lipases are a newly discovered subclass of lipolytic enzymes that can break down lipids and hydrolyze triglycerides into glycerol and free fatty acids (Chepyshko et al., 2012). Thus, far, little data have apparently been published on the detailed role of GSDL esterase/lipase activity in waterlogging tolerance or AR formation. Interestingly, Csa3M669600.1was the highest accumulated (~7.029 fold than control) protein in waterlogged Zaoer-N, and Csa1M058140.1 was induced only in Zaoer-N. These indicated enhanced lipid catabolism, possibly via elevated glycerol to provide additional carbon and energy sources for AR primordia growth or free fatty acids for membrane constituents of those newly developed cells (Li et al., 2016).

Together, this study reveals the proteomic difference of the tolerant line Zaoer-N and the sensitive line Pepino upon exposure to waterlogging stress. Our iTRAQ-based proteomic data showed that the more efficient of regenerate ATP and NAD^+^ and continuation of the glycolysis pathway in tolerant line Zaoer-N appears to be an important way to maintain the level of available carbohydrate and energy to prevent intracellular energy shortage resulting from the waterlogging-caused oxygen depravation. Moreover, the phytohormone ethylene and ROS production might act as the response signaling in activating the apical meristem in AR primordial, whereas the JA might play an opposite role. Compare with Pepino, proteins cell division, cell growth, phenylpropanoid metabolism, and lipid catabolism are also important to facilitate protrusion of the AR primordia to the outside in Zaoer-N hypocotyls. These results indicated that proteomics with bioinformatics analysis is a good starting point for understanding the overall molecular response of cucumber hypocotyls to waterlogging. A deep and broad understanding of the DRPs identified is ongoing to make clear their specific role in ARs emergence upon waterlogging.

## Author contributions

XC conceived the experiment. XX and JJ performed the research. XX, JJ, and XM collected data. XX, QX, and XQ analyzed the data and wrote the manuscript.

### Conflict of interest statement

The authors declare that the research was conducted in the absence of any commercial or financial relationships that could be construed as a potential conflict of interest.

## Supplementary material

Table S1 **Detailed information of the differentially regulated proteins identified by iTRAQ**.

Table S2 **KEEG pathway enrichment analysis of differentially regulated proteins in Zaoer-N**.

Table S3 **KEEG pathway enrichment analysis of differentially regulated proteins in Pepino**.

Table S4 **The abbreviations of the protein names showed in the networks of Figure 5**.

Table S5 **Endogenous jasmonic acid concentrations in hypocotyls of Zaoer-N and Pepino after 2 days of waterlogging**.

Figure S1 **Morphology of 7-day waterlogged Zaoer-N and Pepino**. The water level was kept at about 2 cm above the soil for 7 days and then removed for photography.

Figure S2 **Ratio distributions for the identified proteins**. The changes of protein contents in Zaoer-N **(A)** and Pepino **(B)** 48 h after waterlogging treatment were analyzed. The horizontal axis displays the base Log2-transformed ratios. The red point indicates that the ratio was greater than 1.5, and the green point indicates that the ratio was less than 0.67. The differentially regulated proteins with a fold-change >2 (red points on the right of red lines) or < 0.5 (green points on the left of green lines) in abundance were further analyzed in the present study.
