# Supplementary figures and images for: Comparative Proteomic Analysis Provides Insight into the Key Proteins Involved in Cucumber (Cucumis sativus L.) Adventitious Root Emergence under Waterlogging Stress
Source: Front Plant Sci. 2016 Oct 13;7:1515. doi: 10.3389/fpls.2016.01515 (PMC5062059; doi:10.3389/fpls.2016.01515)

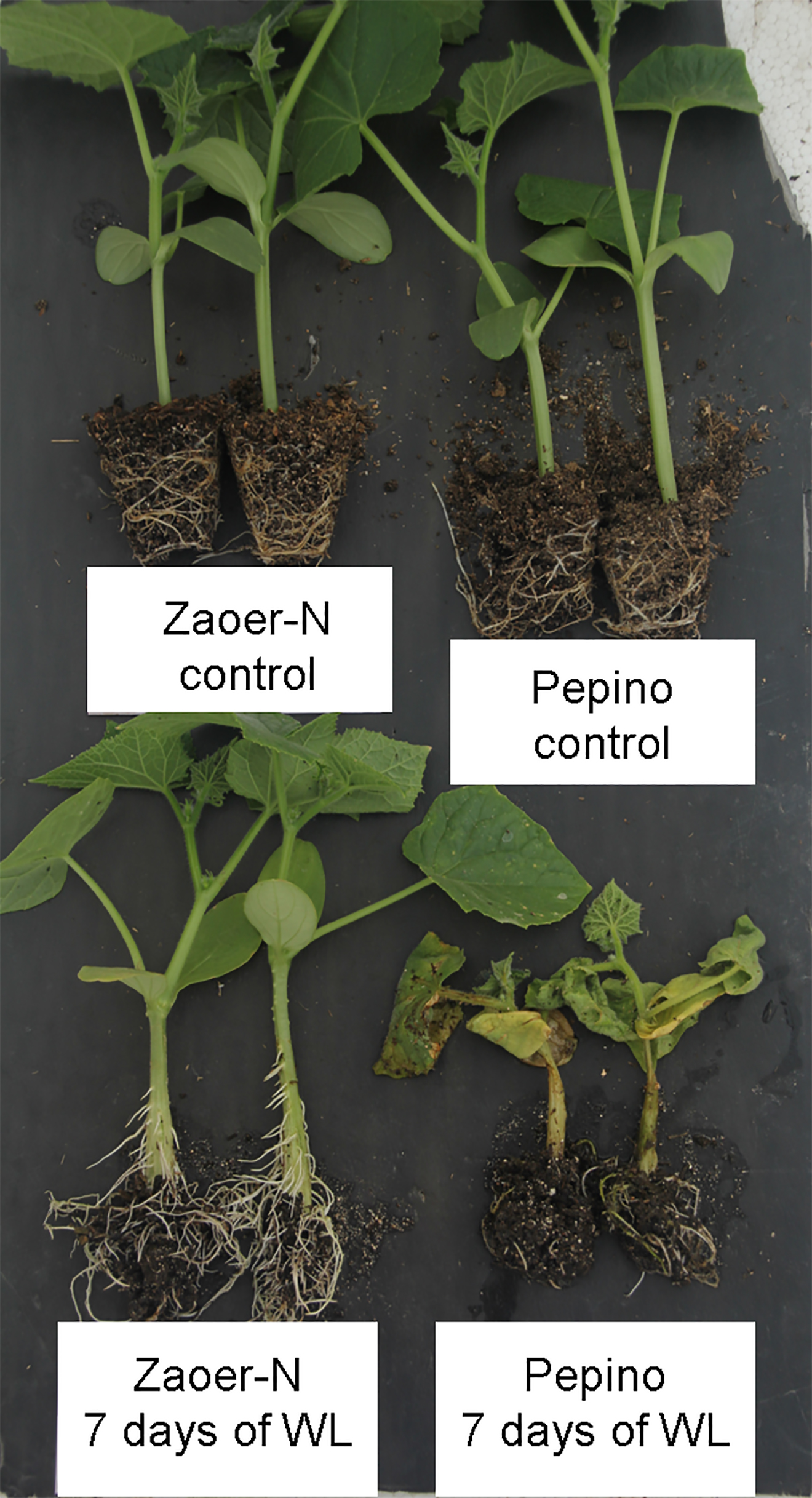

Supplement: Figure S1 — Morphology of 7-day waterlogged Zaoer-N and Pepino. The water level was kept at about 2 cm above the soil for 7 days and then removed for photography. [file Image1.JPEG]

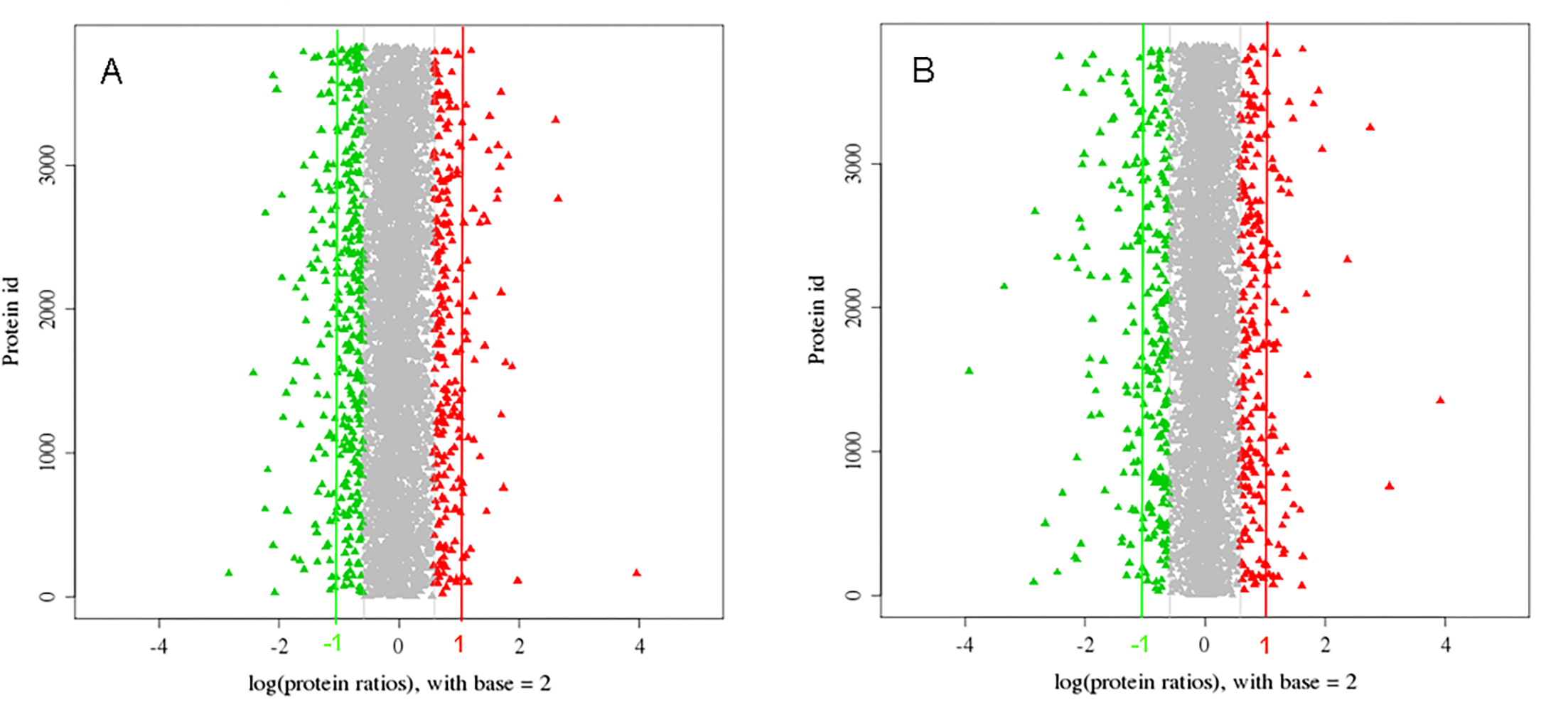

Supplement: Figure S2 — Ratio distributions for the identified proteins. The changes of protein contents in Zaoer-N (A) and Pepino (B) 48 h after waterlogging treatment were analyzed. The horizontal axis displays the base Log2-transformed ratios. The red point indicates that the ratio was greater than 1.5, and the green point indicates that the ratio was less than 0.67. The differentially regulated proteins with a fold-change >2 (red points on the right of red lines) or < 0.5 (green points on the left of green lines) in abundance were further analyzed in the present study. [file Image2.JPEG]
